# Effect of Sfrp5 on Cytokine Release and Insulin Action in Primary Human Adipocytes and Skeletal Muscle Cells

**DOI:** 10.1371/journal.pone.0085906

**Published:** 2014-01-21

**Authors:** Maren Carstensen, Claudia Wiza, Karin Röhrig, Pia Fahlbusch, Michael Roden, Christian Herder, D. Margriet Ouwens

**Affiliations:** 1 Institute for Clinical Diabetology, German Diabetes Center, Leibniz Center for Diabetes Research at Heinrich Heine University Düsseldorf, Düsseldorf, Germany; 2 German Center for Diabetes Research (DZD), Düsseldorf, Germany; 3 Institute for Clinical Biochemistry and Pathobiochemistry, German Diabetes Center, Leibniz Center for Diabetes Research at Heinrich Heine University Düsseldorf, Düsseldorf, Germany; 4 Department of Endocrinology and Diabetology, University Hospital Düsseldorf, Düsseldorf, Germany; 5 Department of Endocrinology, Ghent University Hospital, Ghent, Belgium; Virgen Macarena University Hospital, School of Medicine, University of Seville, Spain

## Abstract

Secreted frizzled-related protein 5 (Sfrp5) is an adipokine with anti-inflammatory and insulin-sensitizing properties in mice. However, the mechanism of Sfrp5 action, especially in humans, is largely unknown. Therefore, cytokine release and insulin signaling were analyzed to investigate the impact of Sfrp5 on inflammation and insulin signaling in primary human adipocytes and skeletal muscle cells (hSkMC). Sfrp5 neither affected interleukin (IL)-6, monocyte chemoattractant protein-1 (MCP-1) and adiponectin release from human adipocytes, nor IL-6 and IL-8 release from hSkMC. In tumor necrosis factor (TNF) α-treated adipocytes, Sfrp5 reduced IL-6 release by 49% (p<0.05), but did not affect MCP-1 and adiponectin release. In MCP-1-treated hSkMC, Sfrp5 did not affect cytokine secretion. In untreated adipocytes, Sfrp5 decreased the insulin-mediated phosphorylation of Akt-Ser473, Akt-Thr308, GSK3α-Ser21 and PRAS40-Thr246 by 34% (p<0.01), 31% (p<0.05), 37% (p<0.05) and 34% (p<0.01), respectively, and the stimulation of glucose uptake by 25% (p<0.05). Incubation with TNFα increased the phosphorylation of JNK and NFκB, and impaired insulin signaling. When Sfrp5 and TNFα were combined, there was no additional effect on insulin signaling and JNK phosphorylation, but phosphorylation of NFκB was reversed to basal levels. Sfrp5 had no effect on insulin signaling in untreated or in MCP-1 treated hSkMC. Thus, Sfrp5 lowered IL-6 release and NFκB phosphorylation in cytokine-treated human adipocytes, but not under normal conditions, and decreased insulin signaling in untreated human adipocytes. Sfrp5 did not act on hSkMC. Therefore, the cellular actions of Sfrp5 seem to depend on the type of tissue as well as its inflammatory and metabolic state.

## Introduction

Chronic, low-grade inflammation in adipose tissue induced by obesity is characterized by an aberrant release of hormones, cytokines and chemokines. These factors affect insulin sensitivity not only in an auto-/paracrine fashion in adipose tissue but also in an endocrine manner in liver and skeletal muscle. Several pro-inflammatory cytokines and chemokines such as tumor necrosis factor α (TNFα), monocyte chemotactic protein 1 (MCP-1) and chemerin, which participate in the detrimental crosstalk between adipose tissue and skeletal muscle and progression of insulin resistance during obesity, were identified [1]-[3]. In contrast, the knowledge about anti-inflammatory cytokines remains limited. Currently, only adiponectin and omentin have been linked to improved insulin sensitivity and are downregulated in obesity and type 2 diabetes (T2D) [4].

Recent studies in mice also suggest an anti-inflammatory and anti-diabetic function for secreted frizzled-related protein 5 (Sfrp5). Sfrp5 antagonizes wingless-type MMTV integration site family member (Wnt)5a in the non-canonical Wnt-signaling pathway [5]. Importantly, Sfrp5-deficiency in mice results in deterioration of high-calorie diet-induced glucose intolerance, hepatic steatosis and macrophage infiltration in adipose tissue. Conversely, acute administration of Sfrp5 to obese and diabetic mice improved glucose tolerance and adipose tissue inflammation [6]. However, one report demonstrated decreased mRNA levels of Sfrp5 [6], whereas others reported increased Sfrp5 expression in obese mice [7]–[10]. Also studies in humans on Sfrp5 yielded conflicting results. In Chinese subjects, both reductions and increases in circulating Sfrp5 levels between obese and T2D patients versus control participants were reported [11]–[13], while no differences were observed between lean and obese Caucasian subjects [14], [15]. Furthermore, Sfrp5 gene expression in adipose tissue was unaffected by obesity [16]. We recently reported a positive association of Sfrp5 with insulin resistance and markers of oxidative stress in mostly overweight and obese Caucasians, indicating that the function of Sfrp5 in humans may be dependent on the subjects' metabolic and inflammatory state [14].

Therefore, the aim of this study was to elucidate the mechanism of Sfrp5 action in primary human adipocytes and skeletal muscle cells (hSkMC) by assessing the impact of Sfrp5 on insulin signaling and release of inflammatory proteins under basal culture conditions and following inflammation-induced insulin resistance.

## Materials and Methods

### Cell culture

Primary human adipocytes were differentiated from cryopreserved human white preadipocytes (PromoCell, Heidelberg, Germany) isolated from subcutaneous adipose tissue from five healthy Caucasian donors (5 females aged 31–58 years). For induction of differentiation, the medium was replaced by PromoCell preadipocyte differentiation medium for 72 h. Then, the medium was changed to PromoCell adipocyte nutrition medium to induce complete differentiation and was replaced every 2–3 days. On day 15–17 of differentiation adipocytes were used for experiments.

Primary hSkMC isolated from the rectus abdominis muscle of four healthy Caucasian donors (2 females, 2 males aged 16–37 years) were obtained as proliferating myoblasts from PromoCell or Lonza (Basel, Switzerland). Differentiation into myotubes was initiated by replacing the growth medium by αMEM containing 2% horse serum (Gibco, Berlin, Germany). Cells were routinely starved on serum-free αMEM on day 6 of differentiation and then used for experiments [17].

Differentiated cells were incubated (i) with or without 10 or 100 ng/ml Sfrp5 (R&D Systems, Wiesbaden, Germany) for 24 h (adipocytes) or 18 h (hSkMC), or (ii) pre-incubated with or without 100 ng/ml Sfrp5 for 4 h, whereafter 5 ng/ml TNFα (Sigma-Aldrich, St. Louis, MO) was added to the adipocyte cultures for 24 h, and 2 ng/ml MCP-1 (PeproTech, Hamburg, Germany) was added to the hSkMC cultures for 18 h. The Sfrp5 concentrations used match those reported in the circulation in human clinical studies [11]–[15]. For analysis of insulin signaling, cells were stimulated with 100 nM insulin (porcine insulin, Sigma Aldrich) (10 min) following cytokine treatment.

### Analysis of inflammation in primary human adipocytes and skeletal muscle cells

Interleukin (IL)-6, IL-8, IL-15, monocyte chemotactic protein-1 (MCP)-1 and adiponectin were measured in cell culture supernatants using Quantikine ELISA kits (R&D Systems). Detection limits for the Quantikine ELISAs for human IL-6, IL-8, IL-15, MCP-1 and total adiponectin were 1.6 pg/ml, 15.6 pg/ml, 2.0 pg/ml, 7.5 pg/ml and 1.6 ng/ml, respectively. Concentrations for all cytokines in cell culture supernatants were above the respective detection limit with the exception of IL-15 for which concentrations did not exceed the detection limit under the described cell culture conditions.

### Analysis of insulin and cytokine signaling in primary human adipocytes and skeletal muscle cells

For insulin and cytokine signaling, cell lysates were analyzed by Western blotting as described [17]. Membranes were incubated with antibodies recognizing Akt-phospho-Ser473, Akt-phospho-Thr308, glycogen synthase kinase 3 (GSK3) α/β-phospho-Ser21/9, proline-rich Akt-substrate of 40-kDa (PRAS40) phospho-Thr246, NFκB-phospho-Ser536, *c-jun* N-terminal kinase (JNK) phospho-Thr183/Tyr185 (all from Cell Signaling Technology, Danvers, MA), Wnt5a or Sfrp5 (both from Santa Cruz Biotechnology, Santa Cruz, CA). All signals were normalized for equal loading by reprobing the membranes with antibodies for glyceraldehyde-3-phosphate dehydrogenase (GAPDH) (Cell Signaling Technology).

### Assay of 2-deoxy-D-glucose (2-DOG) uptake

At day 16 after initiation of differentiation, primary human adipocytes grown in 6-well plates were placed on serum-free adipocyte basal medium (PromoCell) with or without 100 ng/ml Sfrp5. After 24 h, the cells were washed with PBS and then incubated for 45 min at 37°C in buffer containing 50 mM HEPES, 138 mM NaCl, 1.85 mM CaCl_2_, 1.3 mM MgSO_4_ and 4.8 mM KCl [pH 7.4]. Then, cells were stimulated with insulin (100 nM) or kept untreated. After 30 min, 2-DOG uptake was initiated by the addition of 2-deoxy-D-[1-^3^H]glucose (0.5 µCi per well) (Hartmann Analytic, Braunschweig, Germany) in 4 µM 2-DOG. After a 30 min incubation at 37°C, the assay was terminated by two washes with ice-cold PBS, followed by lysis in 1 ml 0.2 M NaOH, 1% SDS. Cell lysates were pH-neutralized by the addition of 12 µl acetic acid, whereafter incorporated 2-deoxy-D-[1-^3^H]glucose was determined by liquid scintillation counting of 900 µl lysate in 5 ml Rotiszint® eco plus (Carl Roth, Karlsruhe, Germany). Data were then corrected for protein content as determined on the remaining lysate using a BCA Protein Assay Kit (Pierce, Rockford, IL).

### Statistical analysis

Differences in cytokine concentrations between various treatments were analyzed by Friedman's test followed by Dunn's multiple comparison test. For analysis of effects on insulin action, the insulin-stimulated condition in the absence of cytokines was set at 100%. Thereafter, differences were analyzed using two-way ANOVA followed by Bonferroni multiple comparison analysis. All data are presented as mean ± SEM. Statistical analyses were performed using Prism 6 for Mac OS X (GraphPad, La Jolla, CA) software. P-values <0.05 were considered as statistically significant.

## Results

### Sfrp5 reduces IL-6 release from human adipocytes but has no impact on myokine release from skeletal muscle cells

**Figure 1:**
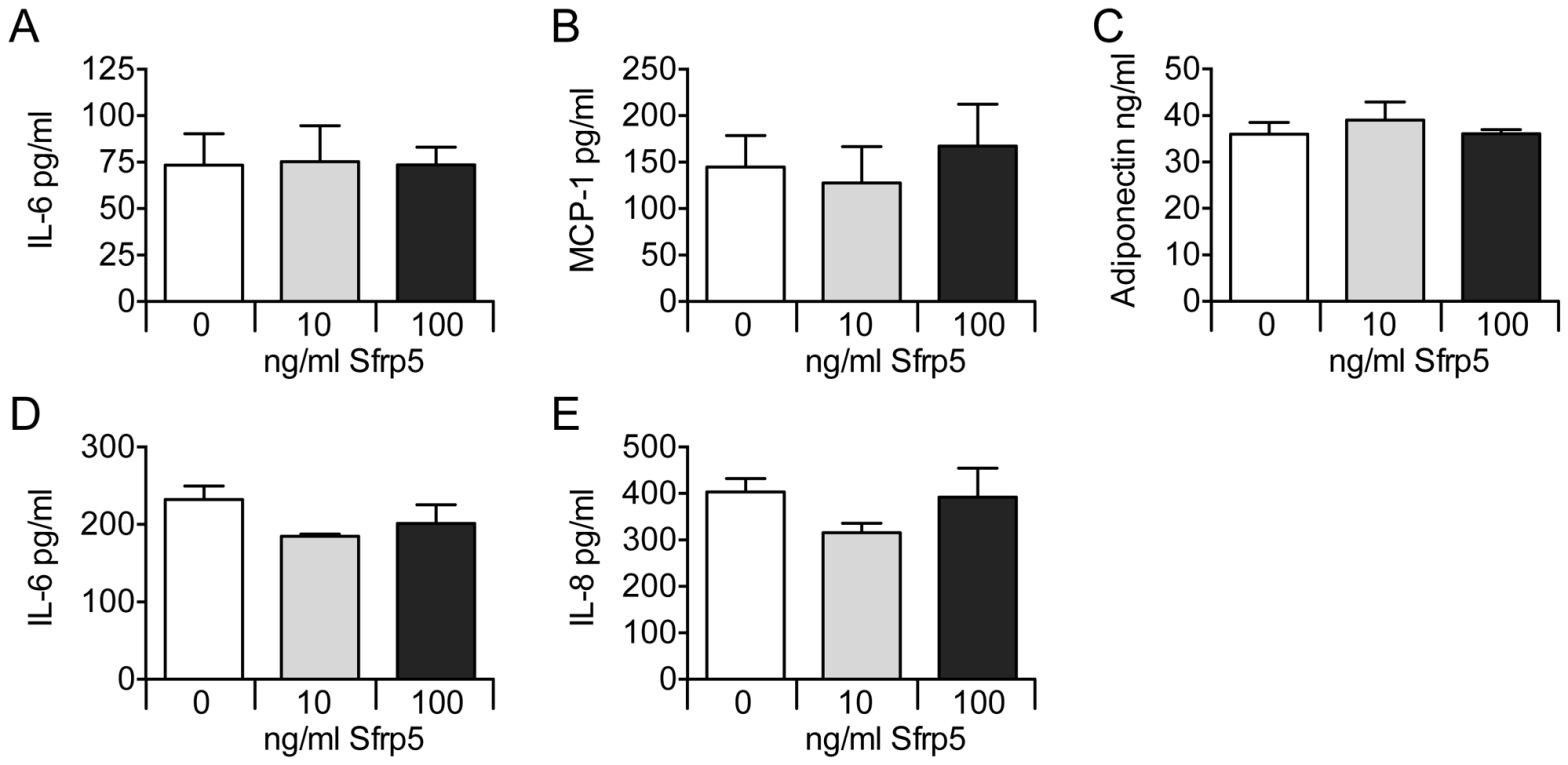
Effect of Sfrp5 on adipokine and myokine release (unstimulated conditions). Primary human adipocytes (A–C) and primary human skeletal muscle cells (D–E) were exposed to increasing amounts of Sfrp5 for 24 h. Cytokine release by the adipocytes and myotubes was quantified by ELISA, and expressed as mean ± standard error of the mean (A–C: n = 5; D–E: n = 4).

**Figure 2:**
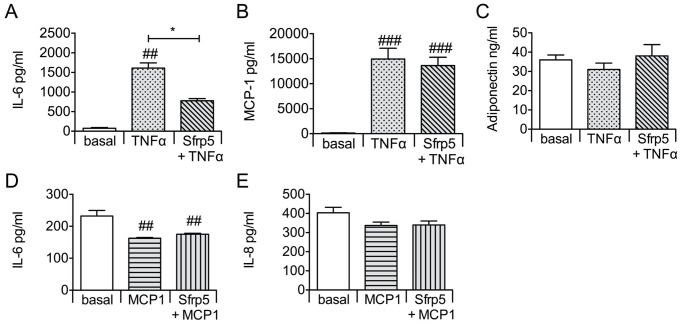
Effect of Sfrp5 on adipokine and myokine release from cytokine-stimulated cells. Primary human adipocytes (A–C) and primary human skeletal muscle cells (D–E) were exposed to Sfrp5 (4 h; 100 ng/ml) prior to incubation with TNFα (24 h; 5 nmol/l) or MCP-1 (18 h; 2 ng/ml). Cytokine release by the adipocytes and myotubes was quantified by ELISA, and expressed as mean ± standard error of the mean (A–C: n = 5; D–E: n = 4). Differences among the various conditions were analyzed by Friedman's test followed by Dunn's multiple comparison test; ### indicates *P*<0.001; ##, *P*<0.01 versus cells kept untreated (basal); *, *P*<0.05 for the effect of the additional Sfrp5 incubation versus TNFα alone.

Under basal culture conditions, Sfrp5 did not affect the release of IL-6, MCP-1 and adiponectin from primary human adipocytes, and of IL-6 and IL-8 from hSkMC (Figure 1). Exposing adipocytes to Sfrp5 prior to TNFα incubation lowered the secretion of IL-6 by 49% (p<0.05), but had no impact on the release of MCP-1 and adiponectin as compared to cells exposed to TNFα only (Figure 2A-C). Preincubation of hSkMC with Sfrp5 prior to incubation with MCP-1 had no effect on IL-6 or IL-8 release (Figure 2D-E). Levels of IL-15 were below the detection limit under all experimental conditions for hSkMC.

### Sfrp5 inhibits insulin signaling and insulin-stimulated glucose uptake in primary human adipocytes

**Figure 3:**
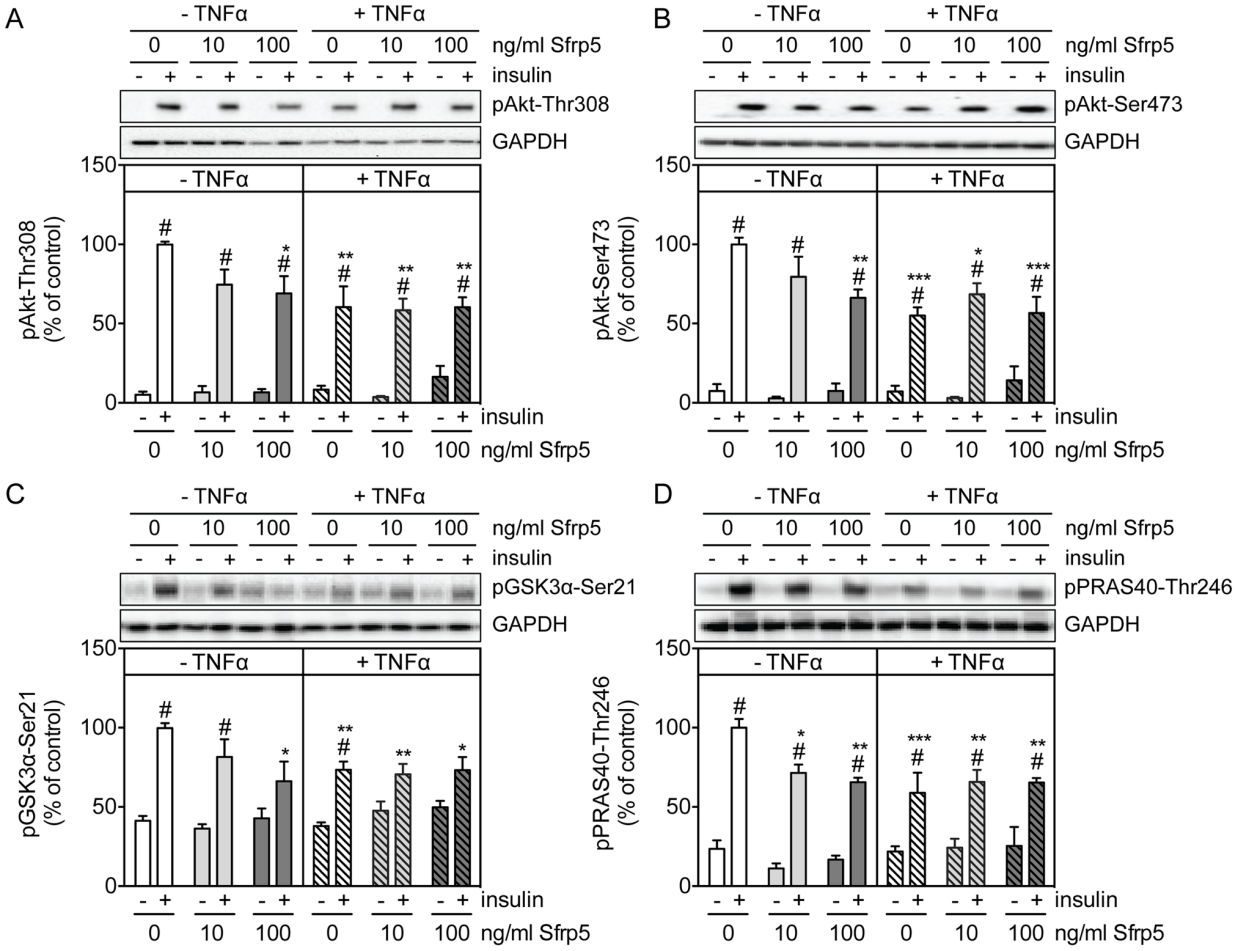
Effect of Sfrp5 on insulin signaling in primary human adipocytes. Primary human adipocytes were kept untreated, exposed to Sfrp5 for 24/ml TNFα for 24 h with or without a 4 h preincubation with Sfrp5. Then, when indicated (+) cells were stimulated with insulin (10 min; 100 nM). Cell lysates were analyzed for phosphorylation of Akt-Thr308 (A), Akt-Ser473 (B), GSK3α-Ser21 (C) and PRAS40-Thr246 (D) by Western blotting. Phosphorylation signals were normalized for GAPDH protein abundance and expressed as mean ± standard error of the mean of five independent experiments using cells from different donors. The values obtained for Sfrp5-untreated, insulin-treated cells were considered as control and set at 100%. Differences among groups were calculated by two-way ANOVA followed by Bonferroni multiple comparison analysis. #, indicates *p*<0.05 for the effect of insulin stimulation; ***, *p*<0.001; **, *p*<0.01; *, *p*<0.05 versus untreated cells.

**Figure 4:**
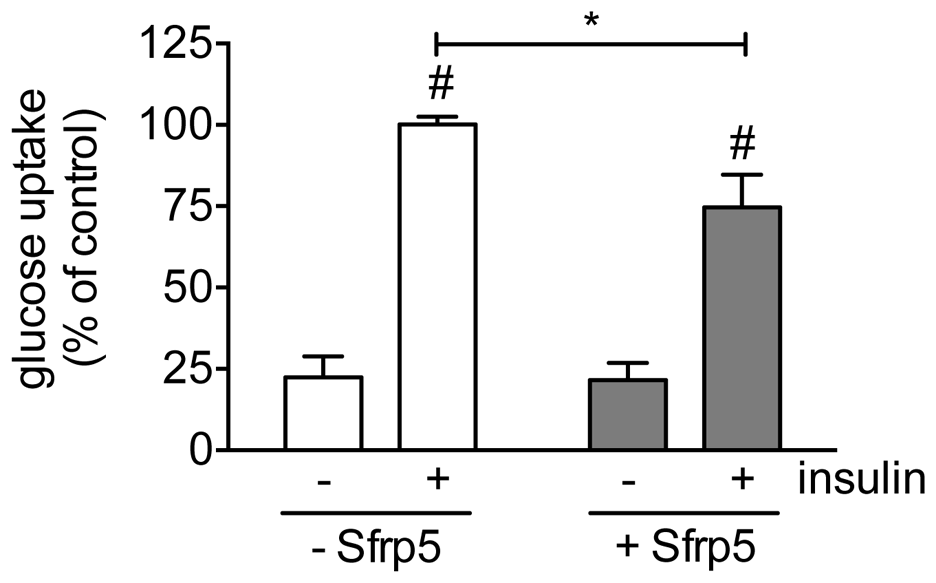
Effect of Sfrp5 on glucose uptake in primary human adipocytes. Mature adipocytes were kept untreated, exposed to 100/ml Sfrp5 for 24 h, when indicated (+) stimulated with insulin (30 min; 100 nM), whereafter glucose uptake was determined. The incorporated amount of 2-deoxy-D-[1-^3^H]glucose was normalized for protein content and expressed as mean ± standard error of the mean of four independent experiments using cells from different donors. The values obtained for Sfrp5-untreated, insulin-treated cells were considered as control and set at 100%. Differences among groups were calculated by two-way ANOVA followed by Bonferroni multiple comparison analysis. #, indicates *p*<0.05 for the effect of insulin stimulation; *, *p*<0.05 for the effect of Sfrp5.

Treating primary human adipocytes with Sfrp5 had no effect on the basal phosphorylation of Akt-Thr308, Akt-Ser473 and its substrates GSK3α-Ser21 and PRAS40-Thr246 (Figure 3A–D). However, Sfrp5 caused a dose-dependent inhibition of the insulin-mediated phosphorylation of Akt-Thr308, Akt-Ser473, GSK3α-Ser21 and PRAS40-Thr246 (Figure 3A–D, left panels). Insulin-stimulated adipocytes incubated with 100 ng/ml Sfrp5 had Akt-Thr308, Akt-Ser473, GSK3α-Ser21 and PRAS40-Thr246 phosphorylation levels that were reduced by 31%, 34%, 34% and 34%, respectively, versus cells kept under basal culture conditions (Figure 3A–D, left panels). Furthermore, Sfrp5 reduced insulin-stimulated glucose uptake by 25% in adipocytes (Figure 4).

**Figure 5:**
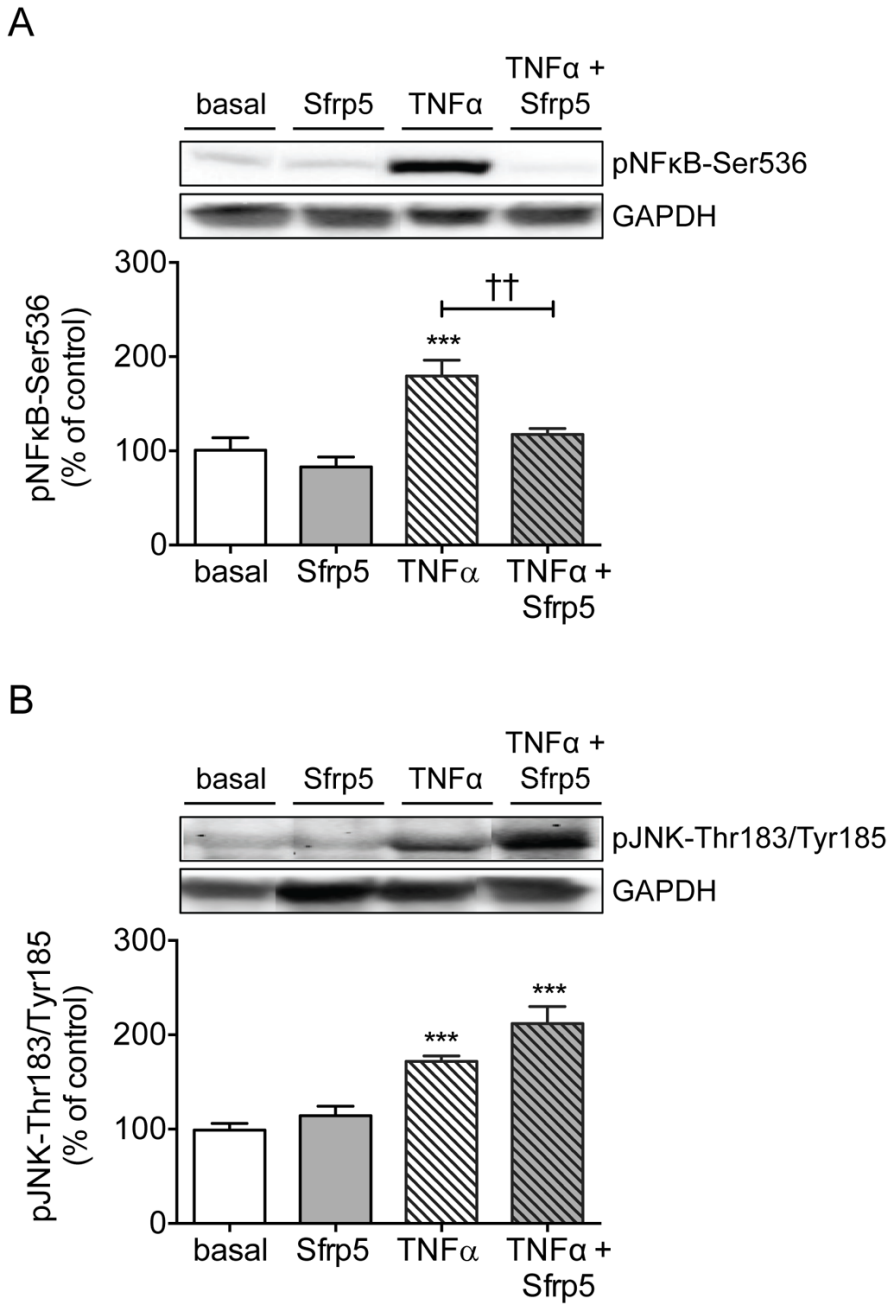
Effect of Sfrp5 and TNFα on inflammation signaling in primary human adipocytes. Primary human adipocytes were kept untreated, exposed to Sfrp5 for 24/ml TNFα for 24 h with or without a 4 h preincubation with Sfrp5. Cell lysates were analyzed for phosphorylation of NFκB-Ser536 (A) and JNK-Tyr183/Thr185 (B) by Western blotting. Phosphorylation signals were normalized for GAPDH protein abundance and expressed as mean ± standard error of the mean of seven independent experiments using cells from different donors. The values obtained for untreated cells were considered as control and set at 100%. Differences among groups were calculated by ANOVA followed by Bonferroni multiple comparison analysis. ***, *p*<0.001 versus untreated cells; ††, *p*<0.01 for the effect of Sfrp5 versus TNFα.

Because some studies ascribed an anti-diabetic and anti-inflammatory action to Sfrp5 [6], [15], we also analyzed the effect of Sfrp5 on insulin signaling in TNFα-treated adipocytes. Incubation of primary human adipocytes with TNFα increased the phosphorylation of NFκB-Ser536 and JNK-Thr183/Tyr185 by 1.8- and 1.7-fold respectively (Figure 5A/B), while reducing the insulin-mediated phosphorylation of Akt-Ser473, Akt-Thr308, GSK3α-Ser21 and PRAS40-Thr246 by 45%, 40%, 27% and 41%, respectively (Figure 3A–D). The phosphorylation of NFκB-Ser536 was reversed to basal levels in adipocytes exposed to Sfrp5 prior to incubation with TNFα (Figure 5A). In contrast, there was no effect of Sfrp5 on the phosphorylation of JNK-Thr183/Tyr185 (Figure 5B). Also, insulin signaling was not restored in adipocytes exposed to Sfrp5 together with TNFα (Figure 3A–D).

### Effect of Sfrp5 on primary human skeletal muscle cells

**Figure 6:**
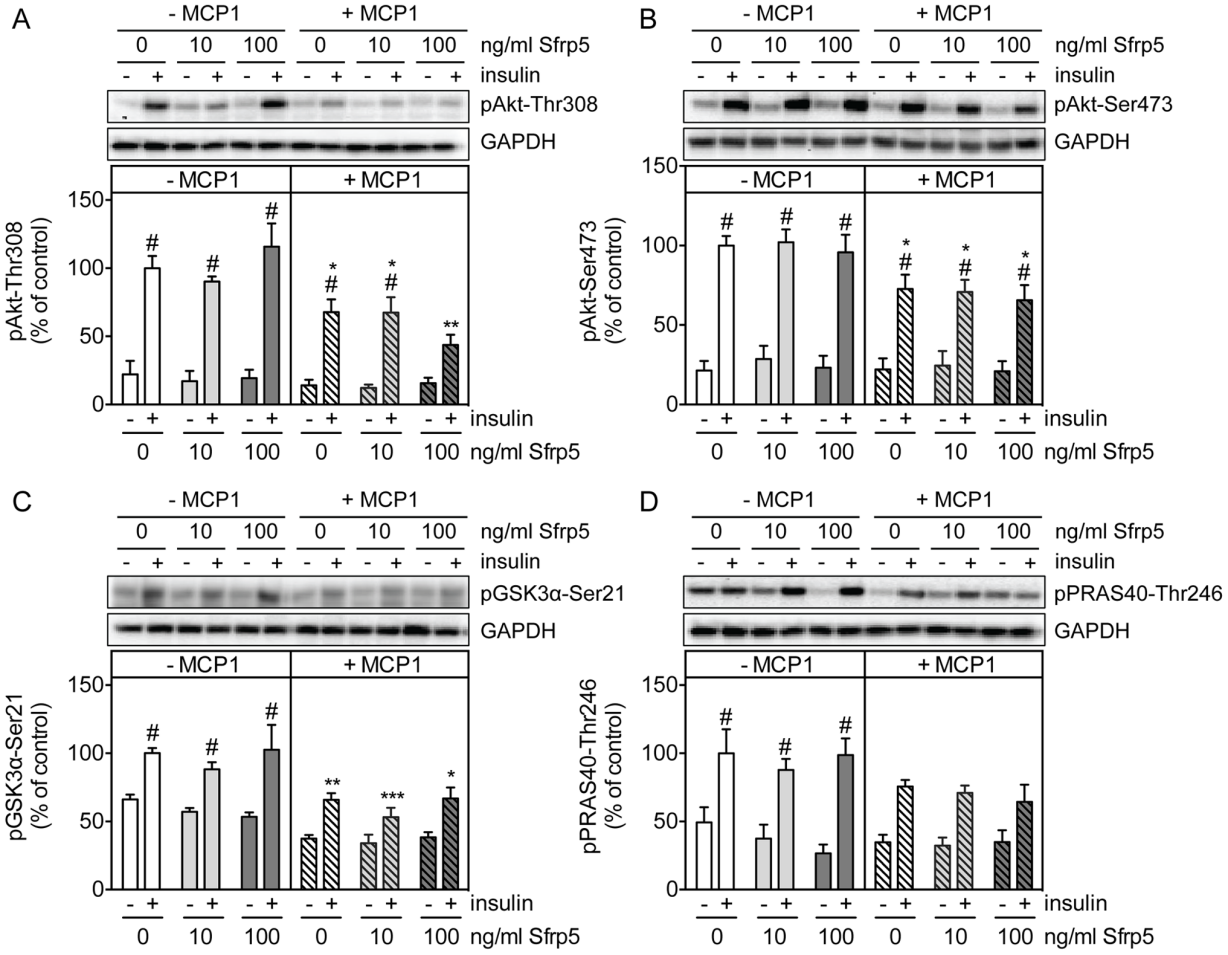
Effect of Sfrp5 on insulin signaling in primary human skeletal muscle cells. Primary human skeletal muscle cells were kept untreated, exposed to Sfrp5 for 24/ml MCP-1 for 18 h with or without a 4 h preincubation with 100 ng/ml Sfrp5. Then, when indicated (+) cells were stimulated with insulin (10 min; 100 nM). Cell lysates were analyzed for phosphorylation of Akt-Thr308 (A), Akt-Ser473 (B), GSK3α-Ser21 (C) and PRAS40-Thr246 (D) by Western blotting. Phosphorylation signals were normalized for GAPDH protein abundance and expressed as mean ± standard error of the mean of five independent experiments using cells from different donors. The values obtained for Sfrp5-untreated, insulin-treated cells were considered as control and set at 100%. Differences among groups were calculated by two-way ANOVA followed by Bonferroni multiple comparison analysis. #, indicates *p*<0.05 for the effect of insulin stimulation; ***, *p*<0.001; **, *p*<0.01; *, *p*<0.05 versus untreated cells.

**Figure 7:**
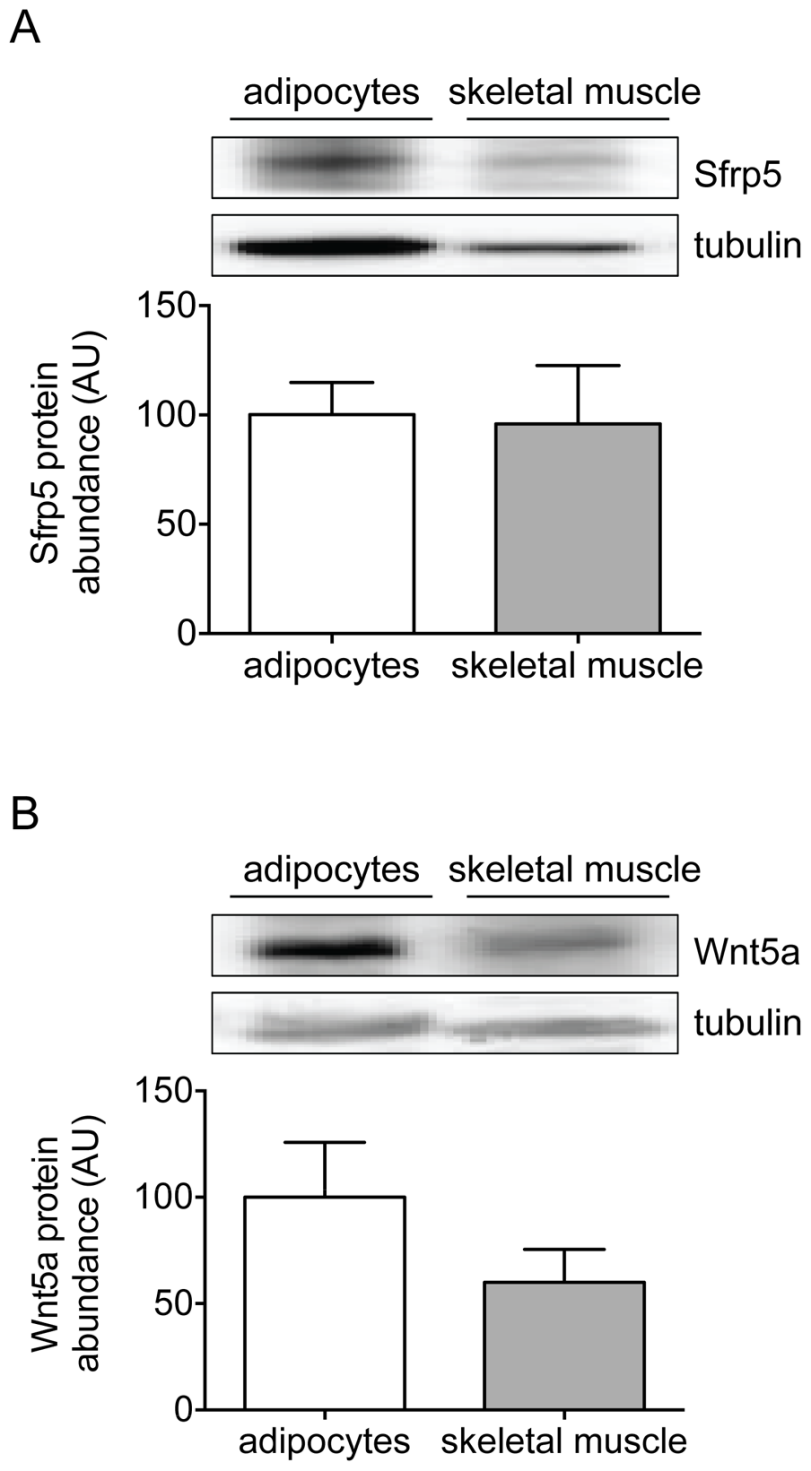
Protein levels of Sfrp5 and Wnt5a in primary human adipocytes and skeletal muscle cells. Lysates from differentiated primary human adipocytes and skeletal muscle cells were analyzed for protein abundance of Sfrp5 (A) and Wnt5a (B) by Western blotting. Phosphorylation signals were normalized for GAPDH protein abundance and expressed as mean ± standard error of the mean of independent experiments (adipocytes; n = 6; skeletal muscle: n = 4) using cells from different donors. The values obtained for adipocytes were set at 100%.

In contrast to primary human adipocytes, Sfrp5 did not affect insulin signaling in hSkMC, neither under basal culture conditions nor following MCP-1-induced insulin resistance (Figure 6A–D). Sfrp5 predominantly affects the non-canonical Wnt signaling pathway by antagonizing the action of the Wnt ligand Wnt5a [5]. While Sfrp5 protein abundance was comparable between primary human adipocytes and hSkMC (Figure 7A), levels of Wnt5a tended to be lower in hSkMC versus adipocytes (Figure 7B). However, even in the presence of recombinant Wnt5a, Sfrp5 neither affected myokine release (Figure S1) nor the insulin-mediated phosphorylation of Akt-Thr308, Akt-Ser473, GSK3α-Ser21 and PRAS40-Thr246 (Figure S2) in hSkMC, thus suggesting that hSkMC are unresponsive to Sfrp5.

## Discussion

The present study shows that Sfrp5 impairs insulin signaling in adipocytes under basal culture conditions. Furthermore, Sfrp5 reduced IL-6 release from TNFα-treated adipocytes. In contrast to adipocytes, Sfrp5 did not act on hSkMC. This suggests that the cellular function of Sfrp5 is tissue-specific and dependent on the metabolic and inflammatory state of the target tissue.

Studies toward the (molecular) mechanism of Sfrp5 action in tissues critical for metabolic control are limited and have yielded conflicting results. Several studies reported the induction of Sfrp5 gene expression during differentiation of 3T3-L1 adipocytes and in rodent models of genetic and/or diet-induced obesity [7]–[10] and propose a role for Sfrp5 in the adipocyte growth via suppression of the Wnt pathway and inhibition of adipocyte mitochondrial metabolism [9]. However, the observed inhibition of IL-6 release and NFκB phosphorylation from TNFα-treated human adipocytes by recombinant Sfrp5 in the present study suggests a protective function for Sfrp5. Interestingly, inhibition of the NFκB signaling pathway was found to prevent the release of IL-6 from human adipose tissue [18]. This protective function for Sfrp5 also fits to the reduction of transcript levels of inflammatory cytokines, including IL-6, observed in adipose tissue from insulin-resistant *ob/ob*, but not wild-type mice, following adenovirus-mediated Sfrp5 expression [6]. Furthermore, a study on Asian subjects with 89 normal glucose tolerant and 87 subjects with T2D found a negative association between plasma levels of Sfrp5 and IL-6 (r = −0.438, p<0.01) [12]. Unfortunately, this study did not mention whether this relation was different between controls and subjects with T2D. A study with a smaller, mostly overweight or obese Caucasian population reported no association between circulating Sfrp5 and IL-6 levels [14]. Thus, more detailed studies involving additional rodent models as well as validation of interaction of Sfrp5 levels with pro-inflammatory cytokines in human clinical samples are clearly required to substantiate the notion that metabolic and/or inflammatory disturbances impact on the mode of Sfrp5 action.

The present study further showed that Sfrp5 inhibits insulin action in primary human adipocytes under basal culture conditions. In support of a role for Sfrp5 in impairing insulin action are data from a clinical study on obese subjects without diabetes in which circulating Sfrp5 levels were found to be associated with HOMA-IR (r = 0.32, p<0.05) [14], and a study on Chinese subjects which reported increased circulating Sfrp5 levels in patients with T2D compared to subjects without diabetes [13]. In contrast, two other studies on Asians showed decreased circulating Sfrp5 levels in patients with T2D versus subjects with normal glucose tolerance, and reported a negative association between plasma levels of Sfrp5 and HOMA-IR (r = −0.446, p<0.01) [12] and (r = −0.444, p<0.001) [11]. Also in mice conflicting data have been reported. One study showed that loss of functional Sfrp5 mitigated increases in serum leptin levels, as well as the induction of glucose intolerance and insulin resistance after high-fat feeding [9]. In contrast, Sfrp5 deficiency led to severe glucose intolerance and further impaired insulin-stimulated phosphorylation of Akt in adipose tissue following high-fat feeding as compared to wild-type mice [6]. This was associated with increased activation of the JNK signaling pathway, which inhibits insulin action via phosphorylation of Ser307 of insulin receptor substrate 1 [19], [20]. In line with observations in 3T3-L1 adipocytes [9], we observed that Sfrp5 had no effect on JNK phosphorylation in primary human adipocytes, neither under basal conditions nor following exposure to TNFα. Therefore, it seems unlikely to ascribe the inhibition of insulin signaling induced by Sfrp5 in human adipocytes to Ser307-phosphorylation of insulin receptor substrate 1. Nevertheless, a limitation of the present study is that we did not examine additional pathways potentially involved in the induction of insulin resistance by Sfrp5, such as activation of the proteasome [17] or the mammalian target of rapamycin complex 1 signaling cascade [21]. The value of such investigations would, however, benefit from recognition of the confounding factors that could explain the conflicting data observed in the *in vivo* studies.

Another aspect that should be considered in this context is that we failed to observe any effect of Sfrp5 on hSkMC. Consequently, the action of Sfrp5 may be tissue-specific. Sfrp5 is known to act as an antagonist for Wnt5a, which in turn activates Wnt signaling through binding to the frizzled receptors [5]. Although Wnt5a is expressed in hSkMC, one may hypothesize that hSkMC do not release Wnt5a, and are therefore unresponsive to Sfrp5. However, we found that even in the presence of recombinant Wnt5a, hSkMC remained unresponsive to Sfrp5 with regard to the parameters examined in this study. This suggests that Wnt5a might not be the only target molecule for Sfrp5 and that there might be other molecules and/or receptors for Sfrp5 associated with its mechanisms of action. For example, Sfrp5 has also been described to antagonize the action of Wnt3a in mouse adipose tissue [9] and of Wnt11 during organogenesis in Xenopus and zebrafish development [22], [23]. However, since Sfrp5 mainly targets the non-canonical Wnt pathway, detailed studies towards the effects of Sfrp5 and/or its Wnt target molecules in hSkMC are currently hampered by a lack of knowledge on the identity of the molecules affected by this non-canonical signaling cascade.

### Conclusion

Sfrp5 attenuated insulin action in adipocytes under normal conditions and reduced inflammation in TNFα-treated adipocytes, but did not act on hSkMC. The mode of action of Sfrp5 in inflammation and insulin resistance might depend on the kind of tissue and defined inflammatory and metabolic circumstances of the site of action.

## Supporting Information

Figure S1 **Effect of Wnt5a and Sfrp5 on myokine release from primary human skeletal muscle cells.** Primary human skeletal muscle cells were exposed to Sfrp5 with (+) or without (−) Wnt5a (10 ng/ml) for 24 h. Cytokine release by the myotubes was quantified by ELISA, and expressed as mean ± standard error of the mean (n = 4).
(PDF)

Figure S2 **Effect of Wnt5a and Sfrp5 on insulin signaling in primary human skeletal muscle cells.** Primary human skeletal muscle cells were exposed to Sfrp5 with (+) or without (−) Wnt5a (10 ng/ml) for 24 h. Then, when indicated (+) cells were stimulated with insulin (10 min; 100 nM). Cell lysates were analyzed for phosphorylation of Akt-Thr308 (A), Akt-Ser473 (B), GSK3α-Ser21 (C), and PRAS40-Thr246 (D) by Western blotting. Phosphorylation signals were normalized for GAPDH protein abundance and expressed as mean ± standard error of the mean of five independent experiments using cells from different donors. The values obtained for untreated insulin-treated cells were considered as control and set at 100%. Differences among groups were calculated by two-way ANOVA followed by Bonferroni multiple comparison analysis. #, indicates p<0.05 for the effect of insulin stimulation.
(PDF)
