# Supplementary figures and images for: Effect of Sfrp5 on Cytokine Release and Insulin Action in Primary Human Adipocytes and Skeletal Muscle Cells
Source: PLoS One. 2014 Jan 21;9(1):e85906. doi: 10.1371/journal.pone.0085906 (PMC3897555; doi:10.1371/journal.pone.0085906)

A

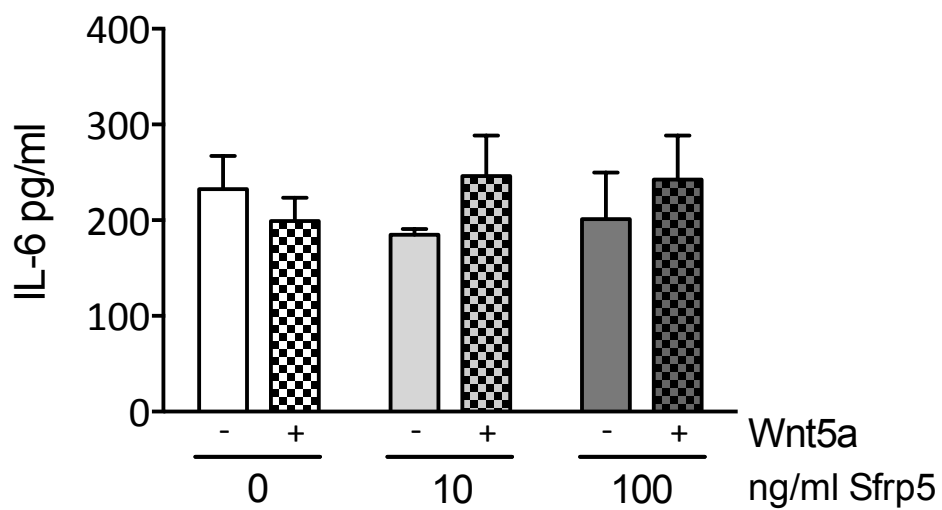

B

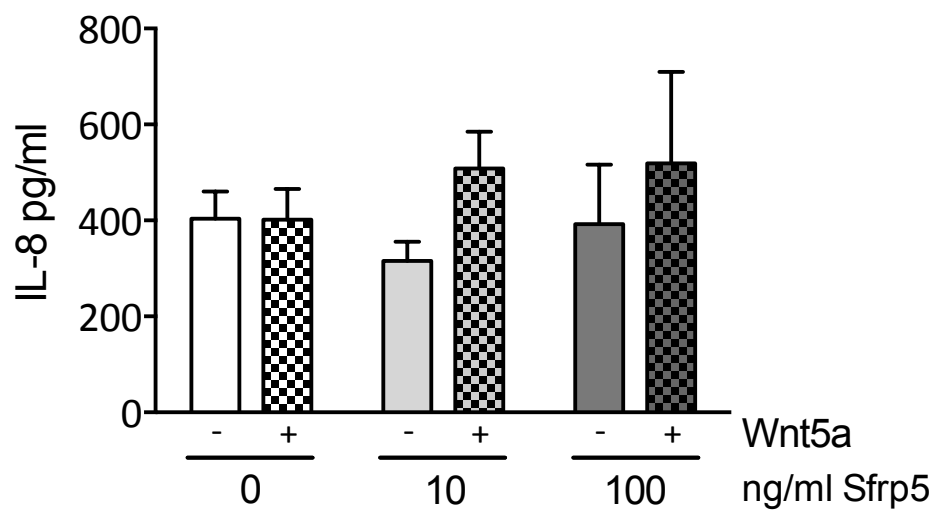

Supplement: Figure S1 — Effect of Wnt5a and Sfrp5 on myokine release from primary human skeletal muscle cells. Primary human skeletal muscle cells were exposed to Sfrp5 with (+) or without (−) Wnt5a (10 ng/ml) for 24 h. Cytokine release by the myotubes was quantified by ELISA, and expressed as mean ± standard error of the mean (n = 4). (PDF) [file pone.0085906.s001.pdf]

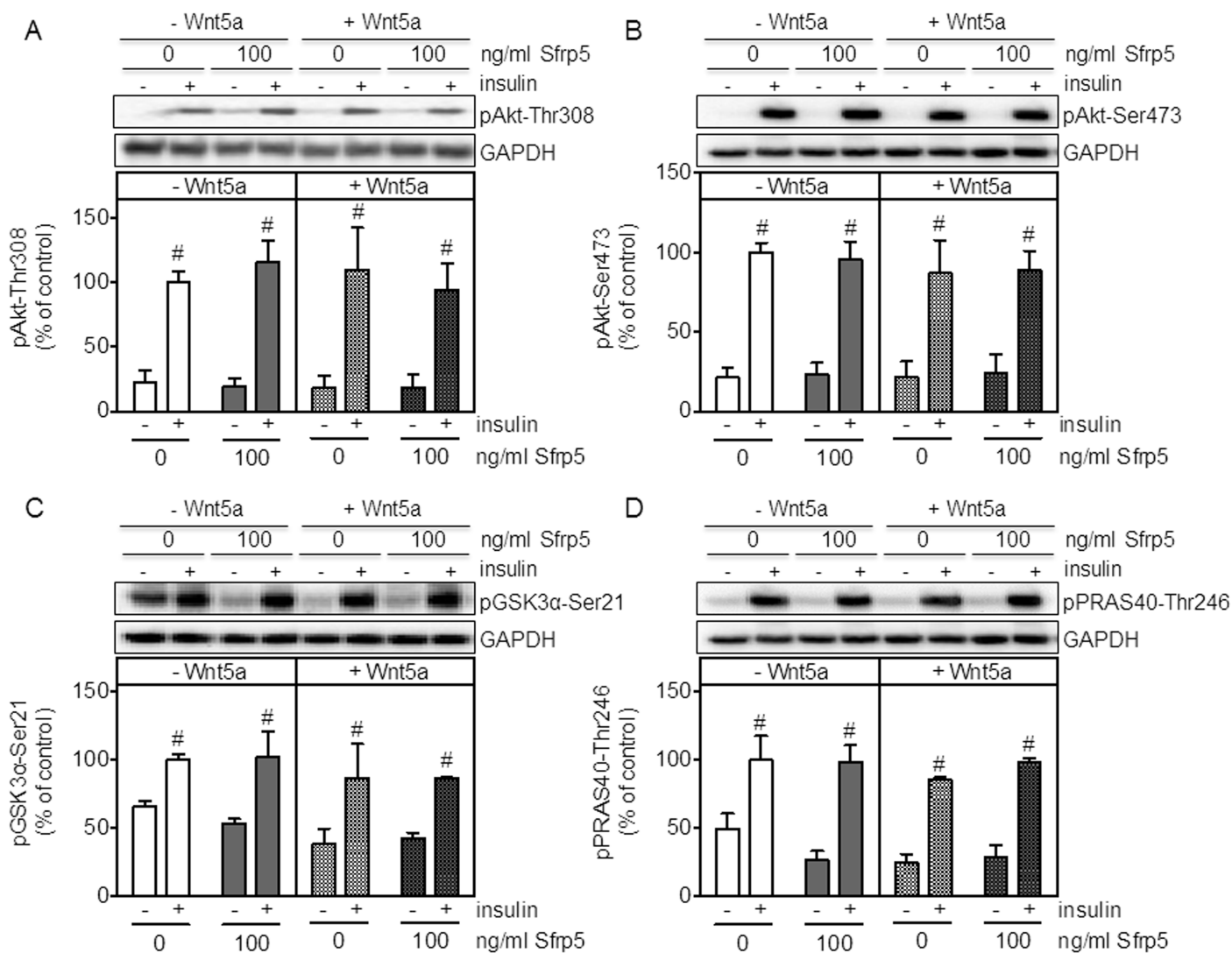

Supplement: Figure S2 — Effect of Wnt5a and Sfrp5 on insulin signaling in primary human skeletal muscle cells. Primary human skeletal muscle cells were exposed to Sfrp5 with (+) or without (−) Wnt5a (10 ng/ml) for 24 h. Then, when indicated (+) cells were stimulated with insulin (10 min; 100 nM). Cell lysates were analyzed for phosphorylation of Akt-Thr308 (A), Akt-Ser473 (B), GSK3α-Ser21 (C), and PRAS40-Thr246 (D) by Western blotting. Phosphorylation signals were normalized for GAPDH protein abundance and expressed as mean ± standard error of the mean of five independent experiments using cells from different donors. The values obtained for untreated insulin-treated cells were considered as control and set at 100%. Differences among groups were calculated by two-way ANOVA followed by Bonferroni multiple comparison analysis. #, indicates p<0.05 for the effect of insulin stimulation. (PDF) [file pone.0085906.s002.pdf]
